# Study on the Reliability and Accuracy of Scolioscope, a New Digital Scoliometer

**DOI:** 10.3390/diagnostics12010142

**Published:** 2022-01-07

**Authors:** Georgios Krekoukias, George A. Koumantakis, Vasileios S. Nikolaou, Konstantinos Soultanis

**Affiliations:** 1 Medical School, National and Kapodistrian University of Athens, 11527 Athens, Greece; vassilios.nikolaou@gmail.com (V.S.N.); ksoultanis@otenet.gr (K.S.); 2 Laboratory of Advanced Physiotherapy, Physiotherapy Department, School of Health & Care Sciences, University of West Attica (UNIWA), 12243 Athens, Greece; gkoumantakis@uniwa.gr; 3 Department of Physiotherapy, Health School, Metropolitan College, 10672 Athens, Greece

**Keywords:** scoliometer, reliability, accuracy, scolioscope, school screening

## Abstract

Early detection of scoliosis with school screening and quick, easy, and reliable assessment of its progress are of paramount importance in the management of patients. There have been several tools described, with the most common being the analog scoliometer. Most recently, smartphone applications have entered this area with and without the use of sleeves for the device. There is no research that has evaluated the accuracy of measurements both left and right in either digital or analog devices. In this study, we evaluated the reliability and validity of a new digital scoliometer called the Scolioscope. Thirty subjects were included for the intra-rater reliability study. ICC values >0.9 were calculated both for same-day and between-day measurements. The device was highly accurate with an average difference from the ones set on the sine bar of 0.03° for right-side measurements and 0.18° for the left. These measurements suggest a highly accurate and reliable tool.

## 1. Introduction

The term scoliosis (scoliosis = crooked) was used by Hippocrates to describe a deformity of the spine. Scoliosis is a three-dimensional deformity [1,2] of the spine with a disorder in the frontal, sagittal, and transverse plane [3]. In the majority of cases (80–85%) the etiology of scoliosis is not known, and the disease is characterized as idiopathic. Idiopathic scoliosis, depending on the age of the patient at the time of diagnosis, is characterized as infantile (0–3 years), childhood (3–10 years), adolescent (10 years-skeletal maturation) and adult scoliosis. The most representative and common form of idiopathic scoliosis is adolescent idiopathic scoliosis with an incidence, according to various studies, in the general population between 0.47% and 5.2% [4]. During the clinical examination for scoliosis, the Adams test is a rough and easy clinical method [5,6]; however, it presents specific weaknesses in terms of its individual application [7].

Simple radiological examination (frontal and sagittal radiographs of the spine) shows three-dimensional deformation over two spinal segments and firstly offers the possibility of direct measurement of the frontal and sagittal deformity, more commonly with the method of Cobb, and secondly allows the indirect assessment of spinal deformation on the transverse plane (rotation of the vertebrae) using various radiological indicators (Lippman-Cobb, Nash-Moe). According to the Scoliosis Research Society (SRS), scoliosis is considered to occur when the Cobb angle is above 10° [8]. Disadvantages of radiological control, for the detection of scoliotic deformities in the general population, is the need for special equipment (radiological machine) with the corresponding financial cost, as well as the exposure of the examinee to harmful radiation [3].

The international literature reports the usefulness of early detection of scoliosis in the student population through school screening [9], resulting in an increase (tripling) of patients who could be treated conservatively in time, and a decrease in those who would re-quire future surgery [10,11]. Therefore, reliable and easy-to-use diagnostic tools are necessary for the clinical evaluation of scoliosis [12].

Various complementary clinical examination tools for the detection of scoliosis have been described in the international literature [13], with the most widely used being the analog scoliometer (or Bunnell scoliometer) measuring the axial trunk rotation (ATR) during the Adams test [14,15,16]. The reliability of the measurements [17] of the scoliometer has been evaluated from very good to excellent [10,16], and the validity of the measurements of this instrument, when correlated with the Cobb angle, found from competent to good [10,18]. That is, the validity of this tool has been evaluated with regard to whether it can detect the presence of scoliosis and not regarding the accuracy of measuring the angle in degrees, which is the definition of validity [19]. It is worth noting that there is no research that has evaluated the accuracy of the scoliometer measurements by comparing them with a given angle, such as from a sine bar or other method. Additionally, the original analog scoliometer (Orthopedics Systems Inc., Union City, CA, USA) which has been researched has a relatively large ball that travels in a tube filled with fluid, and so the actual measurement is open to interpretation [6]. In addition, it is rather expensive and not readily available in most countries.

With smartphones entering the market, many relevant applications have been created, including ones measuring ATR. Only one of those applications has been most researched, the Scoliogauge (Ockendon Partners Ltd., Shrewsbury, UK). There have been studies that assessed this application’s validity without the use of a special phone adapter that has a notch for the spinous process [20,21,22] or with it [23]. These studies suggested that this application is reliable and valid against the scoliometer. However, this application can only be used by an iPhone (Apple Inc., Cupertino, CA, USA), which is influenced by software and hardware updates and therefore cannot be generalized to other electronic devices or similar applications.

There seemed to be a need for a purpose-built device capable of providing clear digital measurements, not influenced by the need of software or hard upgrades. The purpose of this study was the assessment of the accuracy and reliability of a new scoliometer providing digital readouts, called the Scolioscope.

## 2. Materials and Methods

This study had two parts. The first part assessed the accuracy of measurements against a given angle, and the second, the reliability of repeated measurements. The full trial protocol was registered at an international clinical trial database (https://clinicaltrials.gov/, NCT04764136, accessed on 6 December 2021). Recruitment took place between July 2021 and October 2021. The study was approved by the ethics committee of the Attikon University General Hospital (338/1-7-2021) and all volunteers (or their guardians/parents if they were under 18 years of age) signed an informed consent before their participation in the study. This new device has a similar shape to the Bunnell scoliometer, with the difference of presenting the measurement in digital format. The Scolioscope is comprised of an outer shell made of marine plywood that was designed using a three-dimensional design software and built in Greece by G.Kr., using a Computerized Numerical Control (CNC) milling machine. The external dimensions of the shell are 180 × 70 × 15 mm. The digital measuring unit was imported (Shahe, Wenzhou, China) and attached securely to the recess of the shell.

### 2.1. Accuracy Study

**Figure 1:**
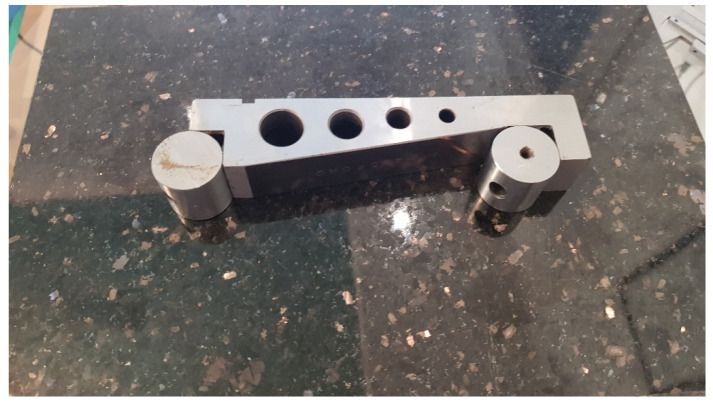
The 5” sine bar and base of granite stone.

**Figure 2:**
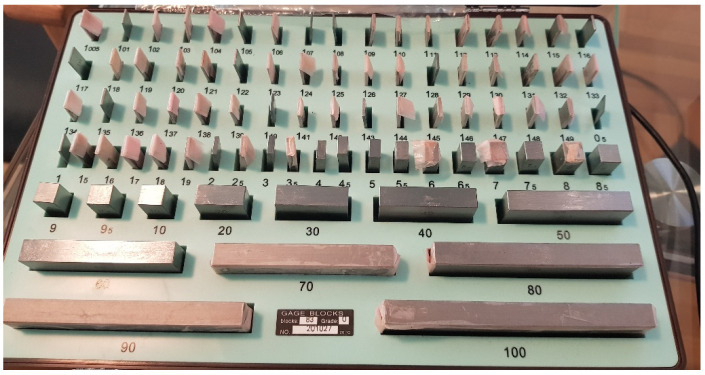
The gauge blocks.

**Figure 3:**
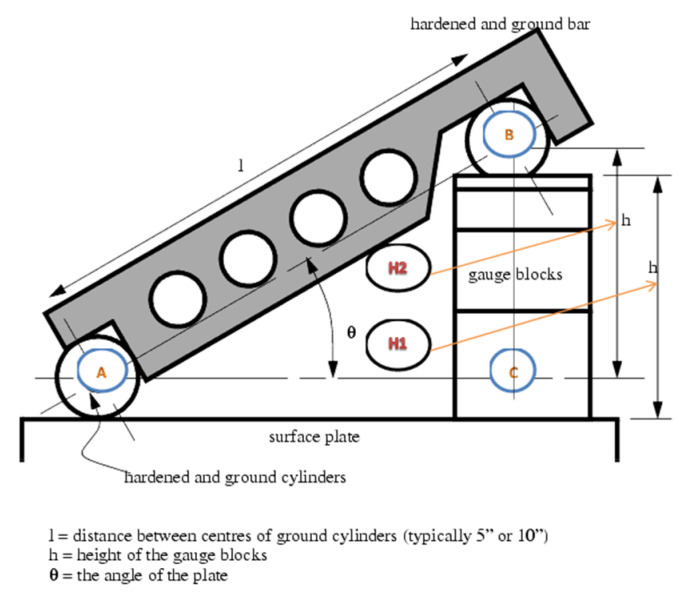
Sine bar with gauge blocks. Adopted from https://openoregon.pressbooks.pub/manufacturingprocesses45/chapter/unit-3-sine-bar/ (accessed on 27 October 2021).

**Figure 4:**
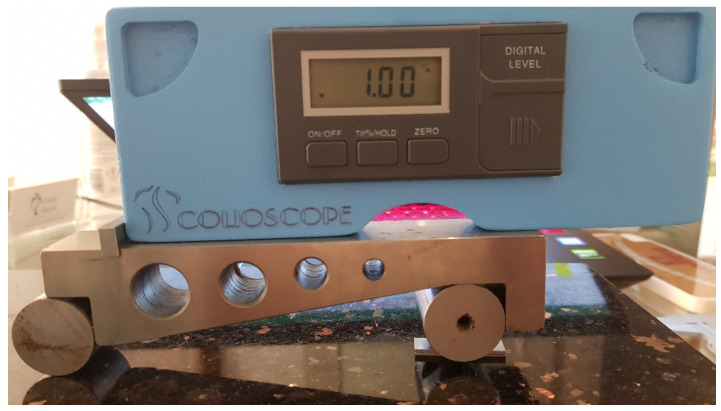
Scolioscope during the accuracy study.

In order to evaluate the accuracy of the measurements, the instrument was placed on a 5” sine bar, which in turn was placed on a 30 by 40 cm, 2” thick piece of granite to provide a stable surface plate (Figure 1). The plate was leveled using a laser level unit (Dewalt DW089K, Leola, PA, USA) and four micro-adjustable feet attached to its underside. Fifteen angle values were randomly selected ranging from 0° to 30°, as this is the range measured by the analog scoliometer. For the randomization process, we used https://randomizer.org/ (accessed 15 June 2021). To set the angle on the sine bar, precision machined gauge blocks (grade 0, Milton tools, Quandong, Figure 2) were utilized that raised the end of the sine bar to the height derived by the formula opposite = sineθΧ hypotenuse (Figure 3 and Figure 4). The angles, their sines, and the calculated opposites can be found in Table 1. The measurements were assessed in both directions (left and right), and this was done three times for each set angle. The average value of the three measurements was measured against the sine bar angle with the use of the t-test and the Pearson correlation coefficient. In addition, the Bland-Altman plot [24] was used to compare the difference between each coupled value.

The process was performed in a temperature-controlled environment steadily kept at 20 °C, as this was the temperature at which the dimensions of the gauge blocks were initially measured, and any other temperature might potentially have minutely changed them and therefore influenced the results [25].

### 2.2. Reliability Study

Power analysis [26] indicated the inclusion of 30 subjects. Inclusion criteria were a cobb angle ≥ 10° and an ability to bend forward. Exclusion criteria were spinal surgery. Demographic characteristics are summarized in Table 2 and full details are given in Table A1 (Appendix A). All participants were recruited from two physiotherapy clinics in Athens, Greece.

One observer at each clinic measured the ATR of participants during the Adams forward bend test [14,15,16]. The observers placed the Scolioscope at the level which they evaluated as having maximal rotational deformation during the test, either on the thoracic or lumbar spine. Both observers were physiotherapists with more than 20 years of experience, five of which were spent assessing patients with scoliosis. Subjects were measured three times at two separate sessions, and the observers chose not to mark the level.

Only the intra-rater reliability was assessed at this time. The Intra-class Correlation Coefficient (ICC) and either the “two-way mixed for consistency” model to evaluate the repeatability of the measurements on the same day (intra-rater reliability), or the “two-way random for absolute agreement” model to evaluate the reliability of measurements at a separate time (test-retest reliability) was used. For the same day, the three measurements were used to calculate the ICC. To determine reliability between days, the mean value of the three measurements from day 1 and the relevant value from day 2 were included to calculate the ICC. The standard error of measurement (SEM) (SEM = √(residual mean square from ANOVA) and the minimum detectable change (MDC) (MDC_95%CI_ = SEM × √2 × 1.96) were also calculated both for the same day and between days.

For all statistical analyses, we used the IBM (Armonk, NY, USA) SPSS software package version 26.

## 3. Results

### 3.1. Accuracy Study

There was no statistically significant difference between the sine bar set angle and the one measured by the Scolioscope (*p* < 0.05) on either the left or right measurements. In addition, the Pearson correlation analysis showed absolute correlation (1) between the actual and average value from both left and right measurements. The average difference between the sine bar set angle and the device was 0.03° (SD 0.03°) for the measurements to the right and 0.18° (SD 0.15°) to the left. This constitutes a highly accurate device.

**Figure 5:**
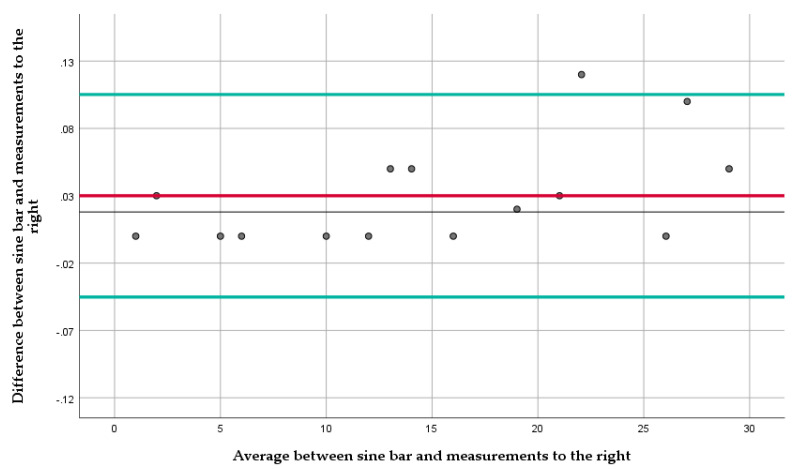
The Bland-Altman plot for measurements to the right. The red line represents the average difference between the sine bar set angles and the mean measurements, and the green lines, the 95% CI.

**Figure 6:**
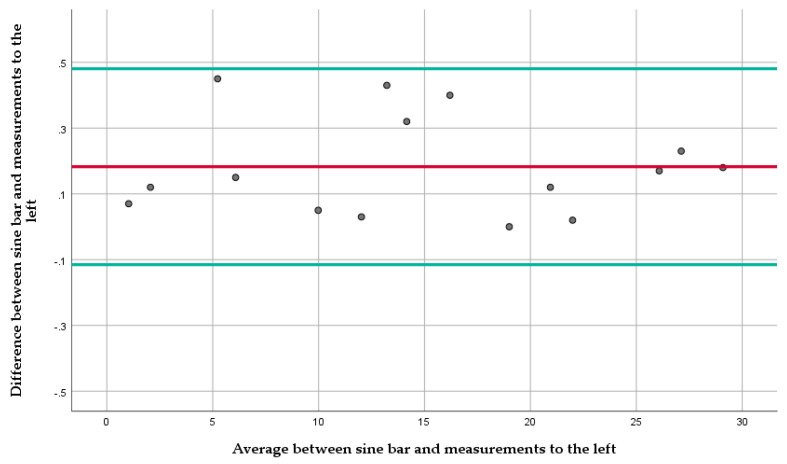
The Bland-Altman plot for measurements to the left. The red line represents the average difference between the sine bar set angles and the mean measurements, and the green lines, the 95% CI.

The Bland-Altman plot for measurements to the right (Figure 5) shows an average difference of 0.03° with a 95% confidence interval (CI) between −0.045° to 0.105°. All measurements fell within the upper and lower limit of the CI, except the measurement at 22° where the difference was 0.12°. The Bland-Altman plot for measurements to the left (Figure 6) shows an average difference of 0.18° with a 95% confidence interval (CI) between −0.11° to 0.48°. All measurements fell within the upper and lower limit of the CI.

### 3.2. Reliability Study

For the same-day measurements, the ICC was calculated at 0.998 (Table 3), which is an excellent ICC value [27]. The residual mean square from the ANOVA table was calculated at 0.029°, and therefore the SEM was 0.17° and the MDC_95%CI_ was 0.472°.

Regarding between-day reliability, the ICC was calculated at 0.997 (Table 4), which again is an excellent ICC value [27]. The residual mean square from the ANOVA table was 0.1°, therefore the SEM was 0.316° and the MDC_95%CI_ was 0.876°.

Data from all individual measurements can be accessed at https://doi.org/10.5281/zenodo.5702015 (accessed on 29 November 2021).

## 4. Discussion

This study provided evidence towards the accuracy and reliability of a new digital scoliometer called the Scolioscope.

### 4.1. Accuracy

Regarding the accuracy of the measurements, the mean measurement difference of 0.03° between the actual and the measured angle to the right and 0.18° to the left are minimal for this type of assessment and below the between-day standard error of measurement of 0.316° (Section 3.2). Measurements to the right were more accurate than ones to the left overall. However, both sides exhibited results within the upper and lower CI boundaries except the measurement at 22° to the right, where the difference from the sine bar set angle was 0.12°, a distance of 0.015° higher than the upper CI boundary and 0.09° from the mean difference. This difference between the left and right measurements, albeit small, could be associated with either the wooden shell forming or the measuring unit whose manufacturer advertised accuracy within 0.2°. In the clinical environment, and especially when measuring ATR, these differences in accuracy would not be considered significant, and therefore, the Scolioscope constitutes a highly accurate tool.

Balg et al. [21] assessed the reliability and validity of a smartphone application (Scoliogauge) to measure ATR. The smartphone (iPhone 4s) was used without an adapter sleeve to cater for spinous process protrusion, and the device was placed directly on the back of the participant. The positioning of the smartphone used without a sleeve, could have especially affected patients with a low body mass index and produced error. They used the Bunnell scoliometer as a gold standard to measure the validity against, and reported excellent correlation between the two (>0.9). The mean difference between the devices was 0.3° for the thoracic spine, and 0.4° for the lumbar. Both the mean difference and the CIs were larger than the ones in this study. However, the results cannot be directly compared, as the authors [21] used the analog scoliometer as the gold standard for which they did not provide evidence as to its accuracy in measuring angles, but rather, its capability in predicting patients with scoliosis.

Guardia et al. [28] assessed the accuracy of two different smartphones (iPhones 4 and 5) using the application Scolioscreen (Spinologics, Montreal Canada), against gauge blocks and plaster casts. They assessed both left and right angles and reported consistent measurements of both devices. However, one of the two (it is not reported which) consistently measured the gauge blocks incorrectly by an average of −1.1 degrees (−0.7 to −1.6). The variability of measuring the casts was greater with a maximum difference of 3.3 degrees. This paper [28] was therefore published as an excerpt from an oral presentation, and due to the lack of further information we could not make a direct comparison of the results. The authors concluded that caution should be used if different phones are used to take measures or if the iPhone is tilted. In addition, both these applications (Scoliogauge and Scolioscreen) were only available for Apple devices, and only those had gone through the rigor of research up to a few years ago. It is unknown whether Android-based applications and devices could provide accurate measurements.

Naziri et al. [29] tried to cover this knowledge gap by testing the accuracy of four different smartphone applications (two on iOS and two on Android) using the sine bar as the gold standard. They used the Scoliogauge and ScoliTrack on an iPhone 5 and Scoliometer and Scoliosis Measurement on a Samsung Galaxy S3. Even though this was not described in Section 2, in the tables of Section 3, a manual (analog?) scoliometer was also included but not otherwise commented on, and it is unclear what it was. The authors [29] did not indicate whether they assessed both directions, and reported mean difference values of −0.14° (SD.31°) for the ScoliTrack, −0.12° (SD 0.12°) for the Scoliogauge, 0.82° (SD 1.52°) for the Android Scoliometer, and 2.26° (SD 1.28°) for the Android Scoliosis Measurement. The iOS applications using the specific iPhone device produced results similar to that of this study when compared to measurements to the left (mean difference 0.18° with SD 0.15°), but less similar when compared to the right (mean difference 0.03°, SD 0.03°). Additionally, these results are better compared to the ones from Balg et al. [21]. The Android applications, on the other hand, produced far less accurate results in comparison to that of this study. The authors [29] did not use any other iPhone or Android device, and according to Guardia et al. [28], this might have influenced the results. Additionally, as the angle increased, so did the mean error. However, this finding was observed and calculated among all four applications, and therefore it is unknown whether a specific device or application was responsible.

Regarding the validity of the analog scoliometer, Amendt et al. [30] assessed the sensitivity and specificity of the device using an angle of 5° or more (5°–10°) of ATR as a cut-off point in determining patients with scoliosis. The authors [30] reported good predictive values but did not comment on the accuracy of the measurements, and therefore, their findings cannot be directly compared to that of this study.

Côté et al. [6] used the Cobb angle as the gold standard. They [6] reported that the scoliometer is more probable to determine thoracic scoliosis, but less probable for lumbar scoliosis than the Adam’s test. The authors [6] concluded that the device had poor precision and inadequate diagnostic accuracy and cannot be used to monitor curve progression due to inherent error, and therefore should not be used as a screening tool. However, they did not measure the actual precision against a given angle, but rather, the ability of the device to predict the existence of scoliosis.

There is evidence to suggest [31,32,33] that an angle of 5° or more (5°–10°) in ATR measured by the scoliometer is necessary to screen for scoliosis in a non-invasive manner. The research available on the analog scoliometer appears inconclusive, and on the smartphone-based ones, is dependent on device and application, but methodological limitations cannot allow generalizability of the results. The accuracy of the Scolioscope™ appears better in comparison to both the analog and application versions, and could therefore be used as a screening tool.

### 4.2. Reliability

The results from this study suggest high intra-rater reliability with ICC values exceeding 0.9. The between-days SEM was 0.316° and the MDC_95%CI_ was 0.876°. Amendt et al. [30] reported high intra-rater reliability of the analog scoliometer. However, the authors [30] chose the Pearson correlation method, which could overestimate agreement [34] and not the ICC, which evidence suggests is better [35]. In addition, the SEM and MDC_95%CI_ were not reported, and therefore, direct comparison was not allowed.

Côté et al. [6] reported excellent inter-rater ICC values for the scoliometer in the thoracic region, and substantial values in the lumbar. They measured the inter-examiner error at 4.9°. The MDC was not reported, and they concluded that the analog scoliometer has poor precision and diagnostic accuracy, and chose the Adam’s test and spinal radiograph to remain the methods of choice to determine patients with scoliosis. As this study examined intra-rater reliability, a direct comparison cannot be made with Côté et al. [6].

On assessing the intra-rater reliability of a smartphone application (Scoliogauge), Balg et al. [21] reported an ICC value of 0.952 from thoracic measurements, and 0.966 for lumbar. These measurements were on the same day, and even though they were excellent, they were still less than the ones observed in this study (0.998). There was no between-days measurement, and the SEM and MDC were also not calculated except the reported CI, which was 2.7°. The Scolioscope™ for the same-day measurements had a SEM of 0.17°, and in comparison, shows a much closer spread of measured values.

The reliability of Scoliogauge was also evaluated by Getnet et al. [22] who used the iPhone 4 to evaluate intra- and inter-rater ICC. The observers did not choose to use a sleeve to place the device in, but rather used their thumbs to balance it on the back of their participants. As the thumb is oval and not round, this might have introduced error in their findings [21]. The intra-rater ICC ranged between 0.871 to 0.932 (depending on the spinal segment) with a mean standard error of 5.97°. Even though the authors did not calculate the MDC, using the formula MDC_95%CI_ = SEM × √2 × 1.96, the result is 16.54°. Although the authors [22] provided evidence towards the excellent intra-rater reliability of Scoliogauge, the high standard error and the derived MDC prove that this application, in combination with the manner used to assess ATR, is not suitable for clinical application [6].

Despite the excellent reliability observed both for the analog and smartphone-based scoliometers, due to the minimal SEM and MDC_95%CI_*,* the Scolioscope™ appears a highly reliable tool.

### 4.3. Limitations/Suggestions

This study assessed intra-rater reliability only. It would be useful to assess inter-rater ICC. In should be noted that the two different observers used two different devices, both of which provided evidence towards the calculation of ICC, SEM, and MDC_95%CI_.

The average BMI was 20.4, which is considered normal for both genders. Perhaps the inclusion of more overweight or obese participants might have influenced the results, and should therefore be considered in another study.

The literature contains controversial data regarding the correlation of the angle of trunk rotation and spinal deformity. There is evidence to suggest a positive correlation between the rib hump and Cobb angle [3,36,37], where others [38] reported that there is no clear linear relationship between the rib hump and vertebral rotation, Cobb angle, and vertebral-rib angle. In a recent study [39], there was strong correlation between the formula predicting the Cobb angle using the scoliometer readings and the actual Cobb angle. Perhaps further research towards correlating Scolioscope ATR measurements and the Cobb angle could provide further insight into the tool’s clinical usefulness.

This device has just been introduced and is not readily available in the market yet. Anyone wishing to perform further evaluation of the device can acquire it by contacting G. Kr. This device, as any new device, also needs to go through the rigor of time and use to determine its robustness and longevity.

## 5. Conclusions

To determine the existence of scoliosis, inexpensive, non-invasive tools and methods with accurate and repeatable measurements are needed. There was a need for a new device capable of being accurate and reliable, and not in any need for a software or hardware upgrade. This study provided evidence towards this, indicating highly accurate and reliable findings of the Scolioscope. Further studies including a larger and more inclusive cohort also assessing inter-rater reliability could provide further evidence towards the clinical usefulness of this new device.

## Figures and Tables

**Table 1:** Angles, sines, and opposites’ calculation for the 5” sine bar.

Angle θ (°)	Sine	Opposite (mm)
14	0.242	30.72
26	0.438	55.67
13	0.225	28.57
27	0.454	57.66
29	0.485	61.57
5	0.087	11.07
16	0.276	35.01
21	0.358	45.51
10	0.174	22.05
6	0.105	13.28
2	0.035	4.43
1	0.017	2.22
22	0.375	47.58
12	0.208	26.40
19	0.326	41.35

**Table 2:** Demographic characteristics N = 30 (22 ♀, 8 ♂).

	Age (Years)	Height (m)	Weight (kg)	Cobb Angle (°)
Average	20.47	1.63	54.33	30.46
SD	13.03	0.07	9.96	7.95
Min	11	1.46	36	15
Max	69	1.79	81	42

**Table 3:** Same-day intraclass correlation coefficient.

	Intraclass Correlation ^b^	95% Confidence Interval		F Test with True Value 0			
		Lower Bound	Upper Bound	Value	df1	df2	Sig
Single Measures	0.998 ^a^	0.997	0.999	1861.683	29	58	0.000
Average Measures	0.999 ^c^	0.999	1.000	1861.683	29	58	0.000

Two-way mixed effects model where the people effects are random and measures effects are fixed. ^a^ The estimator is the same, whether the interaction effect is present or not; ^b^ Type C intraclass correlation coefficients using a consistency definition. The between-measure variance is excluded from the denominator variance; ^c^ this estimate is computed assuming the interaction effect is absent, because it is not estimable otherwise.

**Table 4:** Between-day intraclass correlation coefficient.

	Intraclass Correlation ^b^	95% Confidence Interval		F Test with True Value 0			
		Lower Bound	Upper Bound	Value	df1	df2	Sig
Single Measures	0.994 ^a^	0.988	0.997	344.002	29	29	0.000
Average Measures	0.997	0.994	0.999	344.002	29	29	0.000

Two-way random effects model, where both people effects and measures effects are random; ^a^. The estimator is the same, whether the interaction effect is present or not; ^b^. Type A intraclass correlation coefficients using an absolute agreement definition.

## Data Availability

Data from the measurements can be found at https://doi.org/10.5281/zenodo.5702015 (accessed on 29 November 2021).

## Appendix A

**Table A1:** Characteristics of all participants.

Subject ID	Gender	Age (Years)	Height (m)	Weight (kg)	Cobb Angle (°)	BMI kg/m^2^
1	FEMALE	34	1.65	54	18	19.83471
2	MALE	69	1.67	68	18	24.38237
3	FEMALE	41	1.7	62	22	21.45329
4	FEMALE	11	1.46	50	15	23.45656
5	FEMALE	14	1.58	53	20	21.23057
6	FEMALE	14	1.6	57	31	22.26563
7	FEMALE	12	1.53	36	30	15.3787
8	FEMALE	12	1.52	52	42	22.50693
9	FEMALE	17	1.79	54	32	16.85341
10	FEMALE	15	1.65	54	40	19.83471
11	FEMALE	17	1.69	61	34	21.3578
12	FEMALE	14	1.61	60	42	23.14726
13	FEMALE	12	1.55	41	35	17.06556
14	MALE	33	1.78	81	27	25.56495
15	MALE	32	1.75	77	35	25.14286
16	FEMALE	22	1.69	52	22	18.20665
17	FEMALE	44	1.71	57	34	19.49318
18	FEMALE	32	1.67	60	36	21.51386
19	MALE	12	1.55	41	41	17.06556
20	MALE	14	1.59	58	29	22.94213
21	FEMALE	13	1.56	48	28	19.72387
22	FEMALE	15	1.61	49	32	18.90359
23	FEMALE	16	1.62	51	41	19.43301
24	FEMALE	13	1.53	40	39	17.08744
25	MALE	14	1.59	51	22	20.17325
26	MALE	13	1.58	49	33	19.62826
27	MALE	15	1.64	60	19	22.30815
28	FEMALE	14	1.6	58	28	22.65625
29	FEMALE	15	1.59	55	32	21.75547
30	FEMALE	15	1.63	41	37	15.43152

## References

[1] Weinstein S.L., Dolan L.A., Cheng J.C.Y., Danielsson A., Morcueride J.A. (2008). Adolescent idiopathic scoliosis. Lancet.

[2] Wang W.J., Yeung H.Y., Chu W.C., Tang N.L., Lee K.M., Qiu Y., Burwell R.G., Cheng J.C. (2011). Top theories for the etiopathoenesis of adolescent idiopathic scoliosis. J. Pediatr. Orthop..

[3] Rigo M. (2011). Patient evaluation in idiopathic scoliosis: Radiographic assessment, trunk deformity and back asymmetry. Physiother. Theory Pract..

[4] Konieczny M.R., Senyurt H., Krauspe R. (2013). Epidemiology of adolescent idiopathic scoliosis. J. Child Orthop..

[5] Grossman T., Mazur J., Cummings R. (1995). An evaluation of the Adams forward bend test and the scoliometer in a scoliosis school screening setting. J. Pediatr. Orthop..

[6] Côté P., Kreitz B.G., Cassidy J.D., Dzus A.K., Martel J. (1998). A study of the diagnostic accuracy and reliability of the Scoliometer and Adam’s forward bend test. Spine.

[7] Karachalios T., Sofianos J., Roidis N., Sapkas G., Korres D., Nikolopoulos K. (1999). Ten-year follow-up evaluation of a school screening program for scoliosis. Is the forward-bending test an accurate diagnostic criterion for the screening of scoliosis?. Spine.

[8] SRS/POSNA/AAOS/AAP Position Statement—Screening for the Early Detection for Idiopathic Scoliosis in Adolescents. https://www.srs.org/about-srs/news-and-announcements/position-statement---screening-for-the-early-detection-for-idiopathic-scoliosis-in-adolescents.

[9] Scaturro D., de Sire A., Terrana P., Costantino C., Lauricella L., Sannasardo C.E., Vitale F., Mauro G.L. (2020). Adolescent idiopathic scoliosis screening: Could a school-based assessment protocol be useful for an early diagnosis?. J. Back Musculoskelet. Rehabil..

[10] Coelho D.M., Bonagamba G.H., Oliveira A.S. (2013). Scoliometer measurements of patients with idiopathic scoliosis. Braz. J. Phys. Ther..

[11] Kiely P.J., Grevitt M.P. (2008). Recent developments in scoliosis surgery. Orthop. Trauma.

[12] Kotwicki T., Negrini S., Grivas T.B., Rigo M., Maruyama T., Durmala J., Zaina F. (2009). Members of the International Society on Scoliosis Orthopaedic Rehabilitation and Treatment Methodology of evaluation of morphology of the spine and thetrunk in idiopathic scoliosis and other spinal deformities—6thSOSORT consensus paper. Scoliosis.

[13] Knott P., Pappo E., Cameron M., Demauroy J., Rivard C., Kotwicki T., Zaina F., Wynne J., Stikeleather L., Bettany-Saltikov J. (2014). SOSORT 2012 consensus paper: Reducing X-ray exposureinpediatric patients with scoliosis. Scoliosis.

[14] Fong D.Y., Lee C.F., Cheung K.M., Cheng J.C., Ng B.K., Lam T.P., Mak K.H., Yip P.S., Luk K.D. (2010). A meta-analysis of the clinical effectiveness of school scoliosis screening. Spine.

[15] Sabirin J., Bakri R., Buang S.N., Abdullah A.T., Shapie A. (2010). School scoliosis screening programme-a systematic review. Med. J. Malays..

[16] Sox H.C., Berwick D.M., Berg A.O., Frame P.S., Fryback D.G., Grimes D.A., Lawrence R.S., Wallace R.B., Washington A.E., Wilson M.E.H. (1993). Screening for adolescent idiopathicscoliosis: Review article. JAMA.

[17] Reliability Analysis. https://www.statisticssolutions.com/free-resources/directory-of-statistical-analyses/reliability-analysis/.

[18] Vidal C., Ilharreborde B., Azoulay R., Sebag G., Mazda K. (2013). Reliability of cervical lordosis and global sagittal spinal balance measurements in adolescent idiopathic scoliosis. Eur. Spine J..

[19] Bowling A. (2002). Research Methods in Health.

[20] Franko O.I., Bray C., Newton P.O. (2012). Validation of a scoliometer smartphone app to assess scoliosis. J. Pediatr. Orthop..

[21] Balg F., Juteau M., Theoret C., Svotelis A., Grenier G. (2014). Validity and reliability of the iPhone to measure rib hump in scoliosis. J. Pediatr. Orthop..

[22] Getnet M.G., Jember G., Janakiraman B. (2020). Inter-and intra-observer reliability of scoliogauge app to assess the axial trunk rotation of scoliosis: Prospective reliability analysis study. Int. J. Surg. Open.

[23] Izatt M.T., Bateman G.R., Adam C.J. (2012). Evaluation of the iPhone with an acrylic sleeve versus the Scoliometer for rib hump measurement in scoliosis. Scoliosis.

[24] Gerke O. (2020). Reporting Standards for a Bland-Altman Agreement Analysis: A Review of Methodological Reviews. Diagnostics.

[25] Yanchun L., Zhang Y., Bao W., Wang W., Liu Y. (2021). Temperature Variation of Steel Plate with Different Thickness on Normalizing Process. J. Phys. Conf. Ser..

[26] Arifin W.N. Sample Size Calculator. http://wnarifin.github.io.

[27] Hazra A., Gogtay N. (2016). Biostatistics Series Module 6: Correlation and Linear Regression. Indian J. Dermatol..

[28] Guardia A., Khan M.I., Donauer A., Duke K. (2015). Validation of smartphone inclinometer tools for measuring rib hump in scoliosis patients. Scoliosis.

[29] Naziri Q., Detolla J., Hayes W., Burekhovich S., Merola A., Akamnanu C., Paulino C.B. (2018). A Systematic Review of All Smart Phone Applications Specifically Aimed for Use as a Scoliosis Screening Tool. J. Long Term Eff. Med. Implant..

[30] Amendt L.E., Ause-Ellias K.L., Eybers J.L., Wadsworth C.T., Nielsen D.H., Weinstein S.L. (1990). Validity and reliability testing of the Scoliometer. Phys. Ther..

[31] De Wilde L., Plasschaert F., Cattoir H., Uyttendaele D. (1998). Examination of the back using the Bunnell scoliometer in a Belgian school population around puberty. Acta Orthop. Belg..

[32] Bunnell W.P. (1984). An objective criterion for scoliosis screening. J. Bone Jt. Surg. Am..

[33] Burwell R.G., James N.J., Johnson F., Webb J.K., Wilson Y.G. (1983). Standardised trunk asymmetry scores. A study of back contour in healthy school children. J. Bone Jt. Surg. Br..

[34] Altman D.G., Bland J.M. (1983). Measurement in Medicine: The Analysis of Method Comparison Studies. J. R. Stat. Society. Ser. D.

[35] Koo T.K., Li M.Y. (2016). A Guideline of Selecting and Reporting Intraclass Correlation Coefficients for Reliability Research. J. Chiropr. Med..

[36] Korovessis P.G., Stamatakis M.V. (1996). Prediction of scoliotic cobb angle with the use of the scoliometer. Spine.

[37] Sapkas G., Papagelopoulos P.J., Kateros K., Koundis G.L., Boscainos P.J., Koukou U.I., Katonis P. (2003). Prediction of Cobb angle in idiopathic adolescent scoliosis. Clin. Orthop. Relat. Res..

[38] Thulbourne T., Gillespie R. (1976). The rib hump in idiopathic scoliosis. Measurement, analysis and response to treatment. J. Bone Joint Surg. Br..

[39] Ma H.H., Tai C.L., Chen L.H., Niu C.C., Chen W.J., Lai P.L. (2017). Application of two-parameter scoliometer values for predicting scoliotic Cobb angle. Biomed. Eng. Online.

